# African and Native American foodways and resilience: From 1619 to COVID-19

**DOI:** 10.5304/jafscd.2021.104.008

**Published:** 2021-09-12

**Authors:** Lindsey Lunsford, Melvin L. Arthur, Christine M. Porter

**Affiliations:** a College of Agriculture Environment and Nutrition Sciences, Tuskegee University; 205 Morrison Mayberry Hall; Tuskegee Institute, AL 36088 USA;; b Department of Kinesiology and Health, University of Wyoming; 1000 East University Avenue, Department 3196; Laramie, WY 82071 USA;; c Professor and Wyoming Excellence Chair of Community and Public Health; Growing Resilience Principal Investigator; Division of Kinesiology & Health, College of Health Sciences, University of Wyoming; 1000 East University Avenue, Department 3196; Laramie, WY 82071 USA;

**Keywords:** African American Foodways, Native American Foodways, Food Justice, Ethnography, Restorying, Resilience, Food Systems

## Abstract

The COVID-19 pandemic is flooding and splitting “efficiency” fault lines in today’s industrialized food system. It also exploits centuries of historical traumas, White supremacy, and systemic racism to kill non-White people at triple the rates of Whites.

In 1619, an English ship landed on the shores of the Powhatan confederacy, or, as the English called it, Point Comfort, Virginia. The ship delivered stolen people onto stolen land. This was a first step in founding today’s U.S. food system. Until that time, the people of North America and West Africa had lived off the land for millennia, foraging, hunting, and cultivating food. But 400 years ago, the twin European colonial influences of invasion and enslavement entwined the lives and, to some extent, the foodways of Native Americans and West Africans in what is now the U.S.

Yet, these communities are still resilient. This paper offers re-stories about how African American and Native American communities have adapted and maintained foodways to survive, thrive and renew, from 1619 to COVID-19. Methods include historical and literature reviews, interviews, and brief auto-ethnography.

Even in the face of a pandemic, Native American and African American communities still leverage their foodways to survive and thrive. Some of these food system strategies also illustrate shifts that could be made in the United States food system to help everyone thrive.

## Introduction

This paper tells a story of how African American and Native American foodways have enabled their communities to survive and thrive, even in the face of a pandemic.

African and Native American people have survived enslavement, invasion, and epidemics. From food scraps thrown from enslavers’ tables to commodity foods designed to supplant Indigenous food systems, we created chitlins and fry bread. We made peach cobbler and tempered chokecherry gravy with sugar. We survived by invention and adaptation. Today, African and Native Americans are mobilizing to reclaim, restore, and restory traditional foodways to nourish our cultures, our communities, and the land. Through food, we are reclaiming our health and our heritage.

The resilience of our people and our food systems is now called upon to help us survive the most recent threat to our communities: COVID-19. The virus that causes this disease illuminates and exploits failures of the U.S. public health system, which has put this nation at the top of international illness and death charts. The virus is also exploiting the health disparities resulting from centuries of White supremacy and historical trauma, ravaging African American, Latinx, and Native American communities at rates up to triple that of Whites.

The COVID-19 pandemic is also shining a harsh light on vulnerabilities of the monocultural corporate food system that dominates in the richest countries (and, increasingly, in those they had colonized). Food systems approaches our communities have used to survive four centuries of oppression also offer paths for rebuilding resilience and health in food systems for all in the U.S.

## Background and Methods

### Who

The first author, LL, is a Black scholar activist and professor. I specialize in sustainable food systems and the intersection of racial equity and anti-Blackness in the U.S. food system. I am committed to highlighting narratives that depict how anti-Blackness and resistance to it shaped the development of U.S. food systems and foodways.

My grandmother taught me so many things. Little did I know at the time that the information she imparted would help me survive a global pandemic. Now that she is gone, and in the face of COVID-19, I wish I had listened more. My grandmother’s penchant for canning, cooking, and growing her own food seemed “backward” and old-fashioned to me as a child. Foolishly, I turned my nose up at food preservation, home-cooked meals, and home gardening, three things the world desperately needs now more than ever. That was the food that made us—that made me. From the Trail of Tears to Trayvon Martin, African and Native American communities share long histories marred by loss; loss of land, lives, freedom, culture, and connections to the wisdom of our ancestors. My soul says*, They’ve taken from us in every way. Yet, we endure, and even thrive*. One of the elders I have interviewed insisted I visit her garden so I could pick some turnip greens. If I was writing about these things, she knew I needed to experience them myself, first-hand. I honor her and those greens that nourished me.

The second author, ML, is a scholar in food sovereignty and traditional storytelling, focusing on the Northern Arapaho people. My work is guided by dedication to reparatory justice for Indigenous people and all oppressed populations. We have been denied our ways of life and, for millions of us, our lives. My advocacy is for reparation for this injustice. I pray for change now that is equal to all that has been stolen and lost.

My grandpas always told me stories of the time when the Ghost Dance came to the Wind River Indian Reservation. In spite of the travesties and tragedies behind these stories, they still told them with humor. They told me to only kill what you are going to eat and never waste food. They taught me that our elders and children always eat first. By the time I was born in 1970, two out of three meals were complimentary of USDA commodity foods, or “commods” as we call them. Sometimes we had food for a third meal. We would get hungry, but I always knew that there would be a can of meatball stew or fruit cocktail to be found at my home or at my grandparents’ homes.

The third author, CM, is a White professor who does action research in public health nutrition, food systems, and social justice. I have learned from the content and the standpoint strategies of LL and ML’s restorying work, which they each began as part of their graduate work. I have helped to bridge and braid their restories in the way shared here.

A child of the ‘70s, I grew up on TV dinners, cheese in cans, and English-inspired dinner triads of cheap meat, a frozen vegetable, and a starch. My mother had a garden when I was small. I never helped with the labor, but snatched sugar snap peas, blueberries, and cherry tomatoes when I would pretend to live off the land. As a Peace Corps volunteer in Fiji, I marveled at how my neighbors could grow everything they ate except oils and spices. My sister and I were the first in our family to embrace cooking. My maternal grandmother passed on an oatmeal cookie recipe (whole wheat and wheat germ, no raisins) that I treasure. I only think to mention that because LL and ML both speak of their ancestors. My section here is still full of “I.” But they are teaching me how to tell new kinds of stories.

In this paper, when we say “our,” we mean LL and ML’s larger communities—those descended from West Africans who were enslaved in what is now the U.S. and the Indigenous people who lived here long before the enslavers invaded. We cannot and do not speak for the great diversity within and between them. But we are of them.

### Why

Recent political campaigns have expressed a nostalgic reverence for some of the stories this nation tells about our collective past. For example, an August 2020 campaign mailer from Wyoming’s House representative Cheney promises she will fight to “preserve American history.” As individual co-authors, we read this as a promise, or threat, to prevent the kind of historical narrative we aim to tell here.

With this paper, we are part of a growing body of work that is reclaiming stories of African American and Native American food systems and foodways. This improves the accuracy and fullness of a history that is so often told mainly by enslavers and colonizers. The purpose of this study is to share how the stories and practices of Native and African American foodways could help heal some wounds and build more resilience in the U.S. food system, to help better nourish us all.

### How

Our research is rooted in the rigorous, conventional academic methods of literature review, interview, and, to some extent, auto-ethnography. This paper, however, is not organized according to academic journal traditions. For example, weaknesses in our research and calls for future research appear in this section. Also, we have entwined literature review, results, discussion, and conclusion into one restory.

We used multiple methods in this research. First, LL and ML each developed an extensive restory of African American (particularly Southern) and Native American (particularly Northern Arapaho) foodways, respectively, as part of their culminating graduate research work. ML has published a restory of the Northern Arapaho food system (Arthur & Porter, 2019), and LL is also developing a manuscript that focuses exclusively on African American foodways. We each conducted extensive literature reviews, interviewed community elders and leaders using an open-ended and semistructured approach (*n*=3 and *n*=11, respectively), and analyzed the interviews using a narrative inquiry approach (Arthur & Porter, 2019; Clandinin, 2020; Lunsford, 2020). ML subsequently expanded on his thesis work to include additional interviews and talking circles related to food systems with people in Wind River Reservation, Wyoming. In reading, mentoring, and learning from their independent work, CM increasingly saw strands that connected the two stories.

The co-authors read one another’s work. We researched the disproportionate impacts of COVID-19 on African American and Native American people and on our national food system. We outlined ways that traditional foodways overlapped. We examined ways that movements for food and racial justice offer some solutions to health disparities and weaknesses in the corporate food system. We reexamined our previous research and original data and expanded our literature and news media reviews to encompass these overlaps. We paid special attention to stories and scholarship available in books, because their length enables more complex storytelling, while their accessibility is limited by physical availability and volume.

We turned all of this into the restory you see here (summary in Table 1). It is too long for a conventional journal paper. It is too short to share a compressive history of two complex peoples over more than four hundred years. It is summative and indicative, rather than comprehensive. It does not fully recognize great diversities within and between our communities. It encompasses only the contiguous U.S. states. Stories of Latinx Americans and some Asian Americans often entwine with ours and share many strands (Cohen, 1984; Holmes, 2013), but do not appear here. Stories of these groups often intersect, for example, among those who identify as both Black and Indigenous. We honor and invite restorying research from storytellers of these and other communities, including our own.

## African and Native American Foodways for Resilience

For millennia, West Africans and Native Americans nourished their communities through growing, gathering, and hunting food. Then, Europeans both invaded the Americas and began kidnapping and enslaving West Africans.

In 1619, the first enslaved people arrived in what is now the U.S. Some may have carried seeds in their hair, such as for okra and greens, supplementing the roots and black-eyed peas that their captors transported as food for the Middle Passage journey. In the U.S. South, they grew food for their families in plantation gardens to supplement whatever rations and scraps the enslavers provided.

Native Americans had thrived on these lands, growing corn, beans, and squash; fishing, foraging, and hunting. European invaders forced these communities off their historical homelands and decimated them with epidemics of infectious disease. The U.S. government eventually starved them into concentration camps called “reservations.” But many remembered old foodways and retained, restored, and adapted them to new landscapes.

These are our ancestors. Today, African and Native American communities suffer enormous health disparities rooted in traumas inflicted then and since. However, we also have adapted, developed, and reclaimed our foodways. These have helped us survive and sometimes thrive, even in the face of the COVID-19 pandemic and the additional weaknesses it has exposed in industrialized food systems. This is our re/story of this journey. We have divided our histories into eras (see Table 1). We provide an overview of each era and then, in subsections, illustrate Native American and African American foodways—practices and changes—during that period.

## Living Most of Our Story (From Our Origins to the 1500s and 1619)

For most of human time in North America and West Africa, people lived by hunting, fishing, foraging, cultivating wild foods, domesticating animals, and growing gardens and crops. Each community nourished itself with a different blend of these strategies, based on local ecosystems and cultures.

Coastal ecosystems in both regions provided food in abundance. Conditions for staple crops flourished in the tropical coastal climate (McCann, 2009). In West Africa, as a Portuguese trader in Guinea wrote in the 1600s, “everything necessary for human existence is found in this land in great plenty and sumptuousness” (Carney & Rosomoff, 2011, p. 7). Similarly, an Englishman who lived with Native Americans in the East and Southeast of North America in the late 1700s noted:

Providence has furnished even the uncultivated parts of America with sufficient to supply the call of nature…. If an Indian were driven out into the extensive woods, with only a knife and a tomahawk, or small hatchet, it is not to be doubted but he would fatten, even if a wolf would starve. He could soon start a fire, by rubbing two dry pieces of wood together, make a bark hut, make earthen vessels, and a bow and arrow; then kill wild game, fish, freshwater turtles, gather a plentiful variety of vegetables and live in affluence.

(Adair, 1775, pp. 409–410)

Though some people made contact between the Americas, Europe, and Africa in this period, this did not involve colonization or systemic enslavement (Van Sertima, 1976).

### Native American Foodways

Eastern and southwestern Native American communities farmed, raising what the Iroquois Confederacy describes as the three sisters: maize, beans, and squash. Adair (1775) wrote, “It is surprising to see the great variety of dishes they make out of wild flesh, corn, beans, peas, potatoes, pumpkins, dried fruits, and herbs. They can diversify their courses, as much as the English, or perhaps the French cooks: and in either of the ways they dress their food, it is grateful to a wholesome stomach” (p. 409). He enjoyed, for example, a “wholesome and well-tasted” corn bread, made with bear fat, potatoes and beans (1775, p. 408).

Food writer and historian Linda Berzok (2005) describes six precolonial foodways, adapted to six macro ecosystems. These are summarized below, with particularly distinguishing foods underlined:

- *Northeast Woodlands and Great Lakes region*: Characterized by agriculture, growing the three sisters and vegetables, supplemented with gathering (including indigenous potatoes, nuts, and berries) and hunting (deer, bear, turkey, goose, fish). Specialties include producing maple syrup and sugar and, around the Great Lakes, gathering *manoomin,* or wild rice (pp. 11–12).
- *Southeast*: Anchored by raising maize, with some squash and beans, supplemented by gathering fruits and nuts, fishing, and small-game hunting. Near the coasts, people fished as their primary food supply, especially in what is now Florida (pp. 13–15).
- *Great Plains*: Adapted to the varied climates in this region with both settled agriculture-based foodways and nomadic hunting-gathering ones. In agricultural communities, sunflowers added to the corn-beans-squash mix, along with trade for bison meat. Hunting communities relied on bison and foods they traded and gathered, including *pemmican*, dried and pounded meat mixed with dried berries (pp. 8–9).
- *Southwest*: Developed dryland farming to raise maize, beans, and squash, supplemented with small game and gathering seeds, berries and cactus fruits, and wild greens (pp. 5–7).
- *Northwest coast*: Fished for salmon, with other seafood secondary. Supplemented with plentiful berries, nuts (especially acorns), greens, and lily leaves and roots. Then and now, they share and display this “great food wealth” in potlatches (pp. 7–8).
- *California, Great Basin, and Plateau*: Anchored with shellfish and fish on the coasts, which were always “free of famine” (p. 10). Inland, acorns were a stable and staple food, replaced by pine nuts in the Great Basin, where hunger was common. Small game and invertebrates provided protein (pp. 10–11).

### West African Foodways

Sub-Saharan Africans adapted foodways to three climate zones: the Sahel, Ethiopian highlands, and tropical West Africa (McCann, 2009). Agriculture may have begun in the Sahel, which used to receive more rainfall (Breunig, 2013). Those seeds and farming practices spread to the friendly climates of West Africa and began to anchor foodways there. People grew staples of millet, rice, sorghum, yams, and black-eyed peas (Wallach, 2019).

Then and now, West Africans make spiced stew meals, primarily of vegetables and sometimes augmented with meat and/or fish, served with a preferred starch. Original starch options are African rice, millet, sorghum, and yams (Miller, 2013, pp. 12–13). Later, trade and diffusion from Asia and Mesopotamia made plantains and other rice varieties available, plus fruits such as melons and mangoes (McCann, 2009, p. 25).

In the 1500s, three more options entered West African foodways and quickly became additional anchors in local cuisines: cassava, peanuts, and—especially—maize. Maize was first cultivated from wild grasses by people indigenous to South America. Their seeds and practices spread and were adapted among Native Americans up the eastern coast of North America and in the South, especially the Southwest (Todd, 2008). In the 1500s, the earliest European invaders, enslavers, and traders took corn seeds from South America and brought them to West Africa. Maize proved to be productive and relatively easy to grow and became popular in many communities in West Africa, especially today’s Ghana (Wallach, 2019). Cassava and peanuts followed a similar path. This period also marked a turning point for both Native America and West African communities, with European enslavement, invasion, and encroachment.

### Enduring Enslavement, Epidemics, Encroachment and Invasion (1500s/1619 to the 1700s)

In August 1619, a British ship carrying over 20 enslaved West Africans landed on the shores of the Powhatan Confederacy. About 10 years earlier, the English had invaded the Powhatan area where the ship came to port. They called it Point Comfort, Virginia.

The landing of that ship marks the day that Native and African American foodways met, by force, in North America. It also roughly marks when this nation began building itself into the United States of America, on stolen lands with stolen people. This began inflicting traumas that, today, still reverberate and persist through generations of their children and grandchildren.

#### Dying

Enslavement and epidemics decimated West African and Native American populations. From an African population of about 25 million, at least six million were kidnapped into slavery in the Americas. Many died in the Middle Passage. At least two million more died in the kidnapping raids and wars related to the slave trade, in which some African rulers engaged not only to enrich themselves but also to protect themselves in the face of European colonization and encroachment (Reséndez, 2016). The vast majority were taken to the Caribbean and South America. About 5% were brought to what is now the U.S.

In the same time frame, Native Americans in the eastern coast and islands of North America also endured European encroachment, invasion, and some enslavement. The British invaded most of the coast. The Spanish invaded Florida in 1513, the Southwest in the 1600s, and reached California by 1762, enslaving Native Americans along the way: “Spain was to Indian slavery what Portugal and later England were to African slavery” (Reséndez, 2016, p. 4).

Initially, the deadliest blow to Native Americans was disease imported by Europeans, for which they had little biological defenses. Of the frequent epidemics, the most virulent killer was smallpox, which invaders possibly deployed intentionally in biowarfare (Patterson & Runge, 2002). The epidemics hindered Native Americans’ capacities to gather, hunt, and grow enough food, and fight the invasion (Reséndez, 2016).

#### Suffering Enslavement

For most of the 1600s, the British were struggling to establish their colonies on Native lands, and indentured servants well outnumbered enslaved people in the east. However, by the mid-1700s they had established 13 colonies. They embedded chattel slavery in all of them, but especially in food, tobacco, and fiber production in the South.

For West Africans who survived the Middle Passage to North America, their diversity of languages, communities, and lives were suddenly entwined in one: enslavement for them and the next foreseeable generations of their children. To re-plant their foodways in this new world and life, they depended on strong agricultural skills and memories. They had some okra and greens seeds. They also had the black-eyed peas and yams that their kidnappers stored in ship holds as food for the voyage and which could be planted in their new homes (Carney & Rosomoff, 2009).

What became African American foodways began here, with skills and seeds that West Africans brought across the Middle Passage blending with the foods that their English and then American “owners” wanted prepared for them. They innovated with what was available by region and what little their enslavers chose to make available. They may have also used small-game hunting practices shared by Native Americans (Harris, 2011).

When the enslavers/invaders became American, with the Declaration of Independence in 1776, they doubled down on race-based chattel slavery. In 1787, the group of White “Founding Fathers” drafted the U.S. Constitution with the oxymoronic hypocrisy of declaring, “all men are created equal” with the calculation in which one slave equals only three-fifths of one white man (Article I, Section 2 of the US Constitution, 1787).

#### Fighting to Retain Traditional Land and Foodways

Compared with the kidnapping of people into slavery, changes in foodways and other key aspects of life were more gradual for Native Americans, in pace with European encroachment and invasion. In fact, Native people in the East often shared their food and foodways with the arriving British, enabling the first colonists to survive and, eventually, thrive (Herrmann, 2019).

However, as the British increasingly encroached on their lands with people and cattle, eastern Native Americans fought back. There were three Anglo-Powhatan Confederacy wars between 1618 and 1644, which concluded with the English taking eastern Virginia for good in 1644. The Cherokees fought a war from 1759 to 1761 to keep what is now the Carolinas. Potawatomis, Ojibwas, and Ottawas successfully reclaimed British posts west of the Appalachians in Pontiac’s War. The Iroquois Confederacy mainly continued a strategy of trade and negotiation, with the exception of the Senecas. However, most other eastern Nations turned to war strategies (Herrmann, 2019).

Starting in Florida in the 1500s, Spaniards invaded and enslaved Native people, forcing them into European forms of farming and confined mission living. They then invaded the Southwest in the 1600s (Berzok, 2005). With just a fraction of their population left, Pueblo communities revolted in 1680 and held off the Spanish until 1692 (Romero, 2020). In this period, Diné (Navajo) people chose to begin raising sheep, which Spaniards had imported.

Northwest communities mainly were able to continue traditional life and foodways during this period (Berzok, 2005). People of the Great Plains actually saw some improvement in their access to a key traditional food, bison. As horses and guns arrived much sooner than the invaders who had introduced them, while eastern invasions pushed some Native communities westward, it also equipped them to hunt bison more frequently and successfully (Anderson, 1994; Lowie, 1982; Schilz & Worcester, 1987). Many Plateau and Plains Indian communities who had been farmers became hunters instead (Berzok, 2005).

#### Allying and Being Divided

African and Native Americans had much in common in their foodways, including one-pot stews and using fermentation for food preservation. Native Americans adopted black-eyed peas to such an extent that some mistakenly thought they originated in North America. Corn became a staple among enslaved people and in West Africa. Intermarriage and Natives sheltering people who escaped slavery were common (Miller, 2013). For example, one of the direct relationships Native and African Americans had in this period was via enslaved people in the southernmost colonies/states escaping to Spanish-colonized Florida. Some worked for their comparative freedom by fighting the British and Native Americans on the side of the Spaniards. Some escaped and formed Black Seminole communities near and occasionally with Indigenous Seminoles (Littlefield, 1977). Early contacts also occurred across the Americas because European explorers who often preceded invasion usually brought enslaved servants, usually of African origin, with them (Millner, 2003).

However, threatened by any forms of kinship among those they were aiming to invade and enslave, European colonizers used typical divide- and-conquer strategies to gain and maintain power. These included paying slave-catching bounties to Natives (Harris, 2011), pitting enslaved people who worked in homes against those who worked in fields, and embroiling Indigenous Nations in their wars against other Natives and other European colonizers. For example, tensions remain to this day between the Cherokee Nation and the Cherokee Freedmen who have Black ancestry (Chin, 2014).

Some Indigenous Nations, including Seminole and Cherokee people, also practiced slavery (Blackmon, 2008; Sturm, 1998). At this time people on every continent used some slavery practices, including occasional chattel (inherited) enslavement. What was unique about U.S. institution of slavery was the invention of the concept of “race,” used to claim that some races were less than fully human and to justify enslavement and brutality against those enslaved (Berlin, 1998; Black, 2011). The English colonizers were building what was to become the U.S. economy and society on stolen land using stolen people’s enslaved labor.

### Building the U. S. (to 1865 and the Turn of the 20^th^ Century)

By 1804, all northern states had banned slavery. In 1808, Congress banned any further import of kidnapped West Africans into enslavement. The southern states did not object because they had four million enslaved people already laboring in their fields and homes and the promise of enslaving all their descendants.

Food access remained one of the ways that “owners” controlled enslaved people. The institution and daily practices of enslavement and its related foodways continued, largely unchanged, until the adoption of the 13^th^ Amendment at the end of the Civil War in 1865.

Native Americans in the 19th century suffered violent intensification and formalization of the White American invasion with escalating physical and cultural genocide tactics. As outlined below, this included extensive attacks on and disruption of land access and traditional foodways.

#### Putting Soul into Food

The U.S. economy was built on slavery. The lives of enslaved Blacks and those Whites who were well-off enough to “own” them were deeply entwined, with Whites relying on enslaved people for food production and preparation. Their foodways, then, also entwined, heavily shaped by Blacks. As foodways historian Frederick Opie (2008) observes:

By the nineteenth century, African American foodways displayed corn, rice, greens, pork, and pork seasoned foods, and fried foods. Over time, the planter class took great delight in the dishes of their slaves, such as chitlins; turnip greens, collards, and kale simmered with pork parts; roasted yams; gumbos; hopping John, corn bread, crackling bread, and cobblers and various preparations of wild game and fish.

(p. 36)

According to plantation records and narratives of previously enslaved people, enslavers generally doled out rations of corn and cornmeal with some fatty pork or bacon and milk. For example, Red Richardson, who was enslaved in Texas, recalled, “we ate cornbread, beans, vegetables, and got to drink plenty of milk” (Covey & Eisnach, 2009, p. 18). Who got what and how much varied by plantation, how much an enslaved person labored, and the use of food for reward and punishment (Douglass, 1845).

However, enslaved people gardened, gathered, and hunted small game to supplement their rations. For example, around slave cabins on just one former plantation in Virginia, archeologists found evidence of “pig, cattle, horse, sheep, goat, deer, opossum, rabbit, rat, squirrel, raccoon, chicken, crow, mallard, bird (unidentified), catfish, sturgeon, striped bass, snapping turtle, turtle (unidentified), shellfish, oyster, freshwater mussel, and marine clam” (Covey & Eisnach, 2009, p. 37).

Out of the scraps from animal slaughter that plantation owners would discard, enslaved people also developed chitlins—pig intestines, usually served fried or boiled in a stew. For both Whites and Blacks, chitlins became “a delicacy,” as one elder told LL.

The concept of dessert was imposed on West Africans and their descendants by English and White Americans. They invented soul food dessert mainstays such as sweet bean and sweet potato pies (Miller, 2013; Opie, 2008). They also innovated with cast-off pie crust dough and left over or bruised fruits to create dishes such as peach cobbler (Opie, 2008). During enslavement, however, sugar and desserts were off limits for the people who produced them. Though the idea of dessert was foreign to West Africans, it became part of the culture of their enslaved descendants, starting with molasses with cornbread (Miller, 2013, pp. 240–241). Another foodways shift introduced by the enslavers was the White American view that food “quintessentially meant meat” (Opie, 2008, p. 20). That said, West African use of small amounts of meat to flavor vegetables and stews perseveres to this day in soul food traditions (Opie, 2008, p. 20), such as collards cooked with ham hocks.

#### Negotiating for Survival

Through the 19^th^ century, Confederacies, Nations and Pueblos negotiated for their survival, both practically in daily life and politically, with wars and treaties. Overall, the U.S. strategy was to force Native American people into ever-shrinking portions of North America where Whites had not yet invaded, combined with a secondary approach of assimilation. Forced removal included starving and forcing people onto “reservations,” which included (but is very far from limited to):

- The Indian Removal Act of 1830. President Jackson evicted many Native Americans in the East to lands “granted” to them west of the Mississippi River, mainly in Oklahoma. This further dislocated the foodways of Chickasaw, Cherokee, Creek, Choctaw, and Seminole Nations, who had already adopted many of the agricultural practices pushed by their invaders as a survival strategy. Those who resisted were forcibly marched 5000 miles to Oklahoma on the Trail of Tears; thousands died along the way.
- The Long Walk of 1864. Major General James H. Carleton enlisted the help of Kit Carson in forcing the Dine’ (Navajo) nation out of their Arizona homelands to New Mexico, beginning with destroying their fields, peach orchards, and sheep flocks. As was usually the case with these removals, the new allotted territory was much less fertile than their homelands. Oral history suggests that this was when the Dine’ people invented fry bread, from the flour and lard rations the U.S. provided, to survive.
- Buffalo massacre of 1865–1890. To starve the Great Plains people onto reservations, the U.S. government adopted a policy of destroying their spiritual and physical source of nourishment: the herds of about 30 million bison. As one army colonel exclaimed, “Kill every buffalo you can! Every buffalo dead is an Indian gone!” (Phippen, 2016). Kiowa elder Old Lady Horse said, “The buffalos saw their day was gone. They could protect their people no longer” (Nabokov, 1991, p. 175). By the end of the century, only a few hundred wild bison were left (Phippen, 2016).

The “push” of starvation and violence onto reservations was paired with a “pull” of government- provided food rations for those who complied. These rations included almost entirely nontraditional foods, such as lard, flour, coffee, and beef. Although these rations were always promised, in practice many hungered even on the reservations. A quarter of the people on the Blackfoot reservation in Montana starved to death in the winter of 1884 (Heat-Moon, 2013).

In the 1800s, the U.S. government and Native American Nations increasingly sought treaties to formalize and codify land allocations, foodways access, and other policies such as food and health care provision. Over 500 treaties were signed. The U.S. has broken every one (Deloria, 1985).

The U.S. also deployed four primary assimilation approaches. One was conversion to Christianity. Another was forced removal of children to U.S. government boarding schools, where teachers strove to erase their identities, in addition to subjecting them to starvation and physical abuse. The co-founder of the first of these schools proclaimed, “Kill the Indian, save the man!” (Churchill, 2004).

The two other assimilation strategies directly involved altering foodways. One was to foster or force adoption of European approaches to agriculture, as described previously. The other was the passage of the General Allotment Act, or Dawes Act, of 1887. It enabled individual members of a Native American Nation to individually own and sell “their” federal allotments land to private owners. This created a checkerboard pattern of privately owned “fee hold” lands, often owned by non-Natives, on reservations across the U.S. Today, for example, on Wind River Reservation in Wyoming, Whites outnumber Native people two to one (Census Reporter, 2019).

The century closed with the army’s massacre of hundreds of Lakota people at Wounded Knee, including Chief Sitting Bull. They had been performing the Ghost Dance—a last-chance spiritual intervention created by the Northern Paiute shaman Wovoka to bring back the buffalo and make the invaders retreat (Andersson, 2018).

#### Transitioning to Next Phases of Oppression

African and Native American survivors of these centuries of enslavement and invasion retained threads of traditional foodways and wove them into what was available to nourish their families and communities. They foraged, gathered, and grew what they could. It is possible that some enslaved people exchanged small game hunting and gathering techniques with Native Americans in the South. They also invented survival foods, such as fry bread and chitlins, from what few food resources their colonizers and “owners” provided.

Here, we transition from stories of our ancestors to talk about our most recent generations and communities. This includes switching to using “our.”

The historian Ibram X. Kendi (2016) notes that the term “race” first appeared in a 1606 dictionary, stating that race “means descent … a man, a horse, a dog, or another animal is from a good or bad race” (p. 36). He argues that the British enslavers and invaders used this concept to lump the great diversity of Native Americans and Africans into one group, and not a group they considered “good.” Whites rationalized their own systemic savagery—including chattel enslavement, invasion, whippings, rape, treaty violations, and genocide—by framing their targets as uncivilized, savage, and subhuman. Their justification for chattel slavery and colonization was White supremacist ideals. Rights and principles of equity may be enshrined in law, but not in practice.

#### Reestablishing Dislocated Lives

At the end of the 19^th^ century, the end of enslavement and completion of invasion ushered in new eras for African and Native American people. There was a brief window of hope for Blacks during the 12-year Reconstruction period that followed the Civil War. About 90% of Black Americans had been enslaved and now all were free (Bennett et al., 1993). The end of enslavement brought both great joy and uncertainty to newly freed African Americans, who had been denied literacy or any form of education. Sudden freedom without support—no housing, no food, and only skills they had been allowed to accrue in service of their “owners”—left many at risk of starvation (Harris, 2011). For example, Thomas Ruffin, who had been enslaved in North Carolina, recalled:

We used to dig up dirt in the smokehouse and boil it dry and sift it to get the salt to season our food with. We used to go out and get old bones that had been throwed away and crack them open and get the marrow and use them to season greens with.

(Harris, 2011, p. 138)

By the turn of the century, White supremacy firmly ruled Black lives again. Yet, starting without even bootstraps, we built new lives. We struggled as sharecroppers for White landowners, often with even less food access and little more freedom than during enslavement (Warnes, 2004). In fact, withholding federal food aid was one of many strategies used to force Blacks into sharecropping (Wallach, 2019). Yet we had extensive agricultural skills, maintained and even grown over generations from expertise brought from West Africa to Emancipation. Black Indigenous farmer Chris Newman states, “At the end of the Civil War, nobody was a better farmer than a Black person, especially an emancipated slave in the South” (A Growing Culture, 2020, 9:46).

On lands in Alabama that had once been home to the Taskigi Nation and then a slave plantation, Lewis Adams and Booker T. Washington founded the Tuskegee Institute. Beginning his teaching career with Native American students at Hampton Institute in Virginia, Washington went on to invest in Black agrarian expertise and advocate for Black people to control their futures and livelihoods by controlling their own food systems (Wallach, 2019; Washington, 1907). Thus, the Tuskegee Institute invested in nation-building by helping prepare newly freed people to build new, free lives.

We also began to thrive in places such as Colfax, Louisiana, and the Greenwood neighborhood in Tulsa, Oklahoma (nicknamed “Black Wall Street” by Booker T. Washington). African Americans built entire towns, such as Rosewood, Florida, and Empire, Wyoming. At its peak, Empire boasted 65 farms run by African American farmers using dryland techniques (Rawlings-Carroll, 2019). However, White American supremacists systematically destroyed each of these thriving African American communities via massacres and/or lynchings: Colfax in 1873, Tulsa in 1921, Rosewood in 1923, and Empire throughout its existence from 1908 to 1930 (Brophy, 2002; González-Tennant, 2012; Lane, 2008; Rawlings-Carroll, 2019).

Jim Crow laws and use of the Constitutional slavery exclusion for prisoners meant that violent oppression and some forms of enslavement continued throughout the South (Blackmon, 2008). About six million people fled to the North and West, seeking less oppressive conditions (Wilkerson, 2010). However, as described above, White supremacy was often violently imposed in the U.S. well beyond the South.

Among Native Americans, only about 237,000 of us survived to see 1900. We were primarily scraping out new hungry and despairing lives on reservations (Nabokov, 1991). The U.S. had “reserved” for us the lands least desirable for farming, hunting, gathering, and grazing (Dunbar-Ortiz, 2014). In addition, by 1934 the Dawes Act had led to the loss of two-thirds of even these allotted lands (Nabokov, 1991). Only the Pueblos of the Southwest and Nations of the Northwest remained on fragments of primary historical homelands. However, even that was with extensive encroachment and harm to their foodways, such as dams, broken migration pathways, and pollution. Buffalo Bird Woman, of the Hidatsa people, lamented in the 1920s:

I am an old woman now. The buffaloes and black-tail deer are gone. Indian ways are almost gone. Sometimes I find it hard to believe that I ever lived them. My little son grew up in the white man’s school. He can read books, and he owns cattle and has a farm. He is a leader among our Hidatsa people, helping teach them to follow the white man’s road… But for me, I cannot forget our old ways. Often in summer I rise at daybreak and steal out to the cornfields; and as I hoe the corn I sing to it, as we did when I was young. No one cares for our corn songs now. Our Indian life, I know, is gone forever.

(Nabokov, 1991, p. 182)

Native people became U.S. citizens with the Indian Citizenship Act of 1924. In practice, this added little to our rights or improvements to our plight. For example, Utah and North Dakota did not allow reservation-based people to vote until the late 1950s (Ferguson-Bohnee, 2020). A federal report in 1928 found that we “lived in destitution poverty, and misery” and have “access only to highly deficient education and health services” (Estes, 2019, p. 219). By the 1940s, federal policy was to eliminate us by assimilation. “If you can’t change them, absorb them until they simply disappear into the mainstream culture” (Brown-Pérez , 2017, p. 14) is how U.S. Senator Ben Nighthorse Campbell (Northern Cheyenne) described this strategy. Assimilation included stripping federal recognition of many tribes and adopting the Indian Relocation Act of 1956, which paid moving costs for us to leave reservations for cities. Also, while the South followed a “one drop” rule to identify who to oppress for being Black, the formal policy for Natives was elimination via “blood quantums” deemed insufficiently Indigenous.

#### Example: Facing Food Marketing

White imaginaries of our peoples were also used to contain and constrain us—e.g., “Mammy-ism” (Abdullah, 1998)—while generating profits for corporations, especially food corporations. Three of the most prominent and enduring characters they invented are Aunt Jemima, Uncle Ben, and the nameless Land O’Lakes Indian woman.

**Figure 1.**
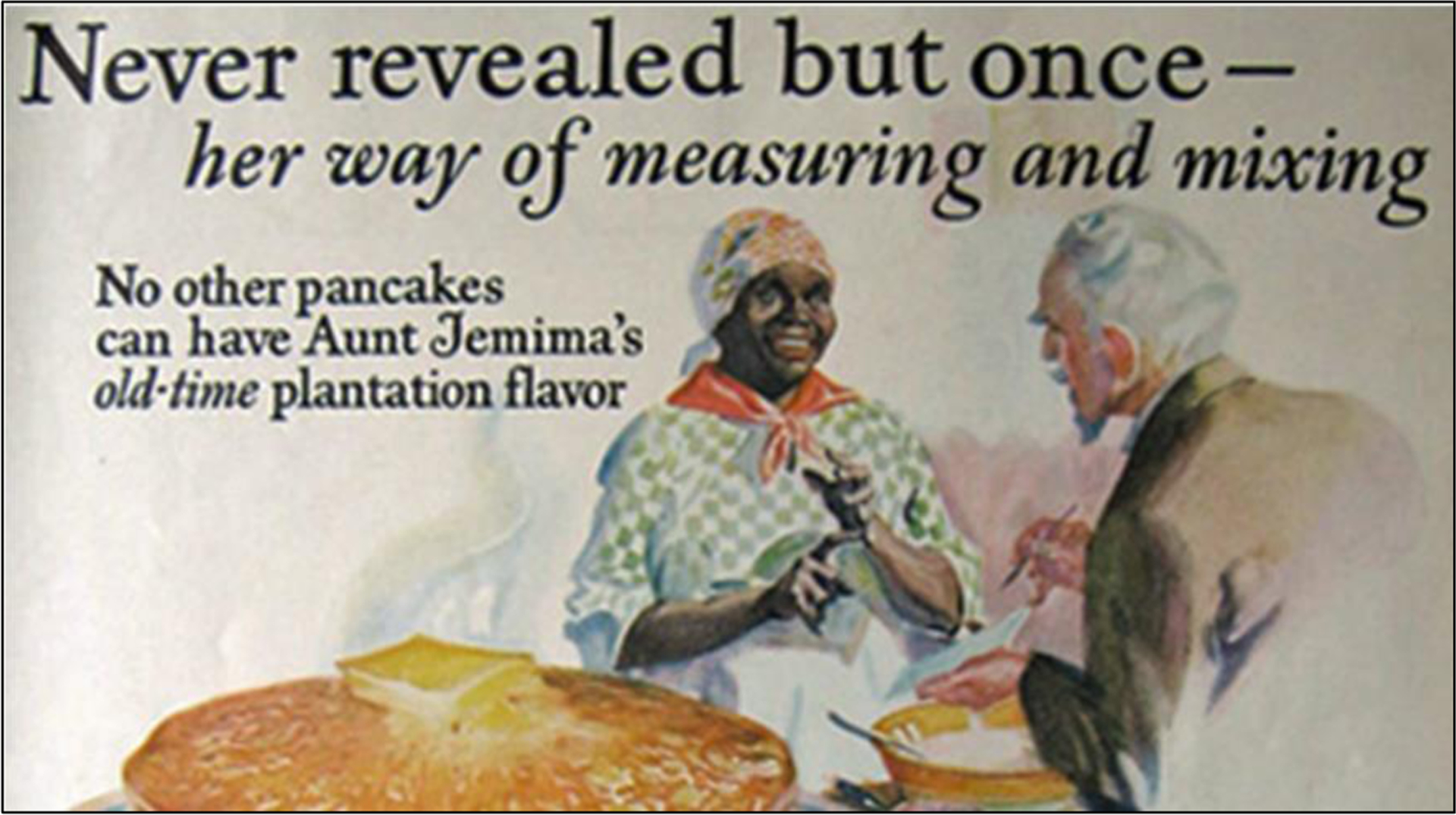
A 1920s Aunt Jemima Pancake Mix Advertisement for “Plantation Flavor” Source: https://namerology.com/2020/06/19/brand-curse-the-name-jemima-in-america/

Aunt Jemima evokes a White ideal of Black women who prepared their food and nurtured their children (often from their own bosoms), whether enslaved or as servants, imagined as done joyfully (Figure 1): “Mammy is the one role White America is still most comfortable with in Black women” (Fuller, 2001, p. 123). Historian Jennifer Wallach (2019) states, in an observation that also applies to Uncle Ben:

When buying Aunt Jemima products, White customers purchased not only tools necessary to make a quick, convenient breakfast, they were also buying into the idea of Black subservience, of a “slave in a box.” The image of a willing Black servant helped assuage White fears of about Black quests for economic advancement and social equality.

(p. 84)

Deployment of White Native American imaginaries was less common in food marketing, showing up more often in sports and tobacco branding. Unlike the ownership and familiarity of the public’s first-name basis with Ben and Jemima, Native imagery is usually abstract and anachronistic. It denotes erasure while fulfilling warrior and/or wisdom fantasies. The Land O’Lakes logo, adopted in 1928, embodies the latter (Heimerman, 2018) (see an artist’s parody in Figure 1).

#### Being Fed Rations and Shame

The modernizing shift in the 1950s to increasingly processed industrial foods eventually spread to all U.S. communities and, more recently, the globe. Euphemistically, this has been called the “nutrition transition” (Popkin, 2017). These foods tend to be high in salt, fat, and sugar, and low in nutrients, contributing to the high prevalence of chronic disease in the U.S. (Boersma et al., 2020).

Such foods arrived early on reservations. First, they were courtesy of federal rations. Then and to the present, the USDA Commodity Supplemental Food Program provides them. Eating these commods, as we call them, yields “commod bods,” with concomitant disproportionate morbidities and death rates (Vantrease, 2013). Native Americans invented frybread out of the salt, flour, and lard provided in original rations. This survival food has become embedded in today’s Native American cultural foodways. Now that most Americans—of every racial group—are eating more than enough calories and fats, it would be hard to argue that frybread is part of a healthy diet from any biological standpoint. Because of its negative health impacts and colonization origins, some Native food sovereignty leaders suggest reconsidering frybread’s role in Native foodways today (Mihesuah, 2016).

Shaming of soul food, i.e., African American foodways (Henderson, 2007; Nettles, 2007; Rankins et al., 2007), adds insult to these injuries. For example, CP recalls an African American presenter at a public health conference saying he no longer eats watermelon, though he loves it, because he cannot dissociate it from a lifetime of white racist taunting about the fruit (see, e.g., Black, 2014). Fried chicken has been similarly deployed in racist tropes. For example, a newspaper columnist describes her struggle to reclaim the food’s African American history along with her love of eating it, writing, “that we’ve been bullied and made to feel ashamed of it is one of the biggest outrages in culinary history” (Thompson, 2020, para. 29). Traditional Native foods and soul foods offer the original slow, local, farm- and forest-to-plate foods now venerated by foodies and nutritionists. All original Indigenous food and traditional vegetable-based West African stews are nutrient-rich. Enslaved people invented fruit- and vegetable-based desserts in order to satisfy White sweet tooths. Prior to enslaving and colonizing, the English elite had viewed leafy and root vegetable staples— which anchored the diets of Black, Native, and poor Whites—as lowly food of the poor. This resulted in diet-related illness such as gout among the English ruling class (Opie, 2018). Without irony, one nutrition study calls for “modifying traditional soul foods” by suggesting stews that are “heavy on vegetables and light on meat” (Rankins et al., 2007, p. S9). Overall, many foodways that West Africans and their enslaved descendants brought to the U.S. have been embraced as general Southern and American foods, ignoring their roots in African culture (Deetz, 2017).

In addition, the foundational food for much of Native America—corn—has been bred and processed into lower-nutrient, homogenous, genetically modified forms. Corn in the U.S. now serves mainly as an ingredient in highly processed foods, feed for industrialized beef and pork production, or as fuel rather than food.

Overall, the “nutrition transition” for our communities has been magnified by decades of supplanting traditional foods with commodities and the heavy marketing of fast food to African American communities (Demby, 2014). African and Native Americans suffer higher rates of chronic stress, substantially caused by racism and disproportionate food insecurity and poverty. This amplifies the effects of poor diet on chronic disease (Bale & Jovanovic, 2020; Gregory & Coleman-Jensen, 2017; Teufel-Shone et al., 2018).

Overall, for us, food is more than nutritionism (Scrinis, 2008). As one African American elder and foodways expert said to LL:

Once they stop you from doing it and carrying on with soul food, you ain’t got nothing left. What you got left? They taking everything away from you. So you just got to keep … you keep. Just like they keep they Confederate flag…. We’re gonna keep our soul food.

Even if not always biologically nutritious, traditional foodways and survival foods can be a nourishing source of healing, comfort, and wholeness for us.

### Reclaiming and Restoring (1960s–2000s)

Food has always played a role in oppressing us, but also in our resistance and reclaiming lifeways and foodways. Starting in the late 1950s, resistance movements in our communities gained people and power, including via foodway strategies.

#### Organizing with Food and Foodways

Food and foodways are threaded throughout our fights for justice and sovereignty. One strand has been simply the logistics of feeding the front lines. This includes, for example, decades of cooking by and for Black civil rights organizers (Schute, 2012), the Black Panthers inventing school breakfast programs (Gebreyesus, 2019), protesting Jim Crow laws by sitting at lunch counters, air-dropping food to the Wounded Knee occupation (Levin, 1998), and setting up kitchens to feed Standing Rock protesters starting in 2016 (Estes, 2019).

Another strand is fighting for access to food and foodways, which includes the lands and waters that nourish us. This involves extensive and ongoing legal battles for honoring treaty obligations for access to lands, waters, and traditional food sources. A recent major win, *McGirt v. Oklahoma*, restored half of that state to the Creek Nation, belatedly honoring an 1833 treaty. The struggle also includes securing compensation for decades of inequitable USDA services to our farmers, via winning *Pigford vs. Glickman* and *Keepseagle vs. Vilsack*. Some funding from the latter has been used to establish the Native American Agriculture Fund,^1^ which is being invested in Native American food sovereignty projects. Its list of grantees^2^ serves as a map of the healing and restoration work in Native foodways across the country.

The central warp for the weft of these threads is continuing, recovering and reclaiming our foods and food traditions. Our communities have led these efforts since at least 1619, with initiatives such as White Earth Land Recovery Project^3^ (founded 1989) and Detroit Black Community Food Security Network^4^ (2006). National food justice and sovereignty organizations began forming in the 1980s. Among Native Americans this includes the First Nations Development Institute^5^ (founded 1980), Intertribal Agriculture Council^6^ (1987), Indian Land Tenure Foundation^7^ (2002), Indigenous Food and Agriculture Initiative^8^ (2013), Native American Food Sovereignty Alliance^9^ (2014), and NCAI’s Tribal Food Sovereignty Advancement Initiative^10^ (2019). African American national organizing groups, with founding dates where available, include the National Black Farmers Association^11^ (1995) and many other landownership retention organizations, Southeastern African-American Farmers Organic Network^12^ (2006), Black Urban Growers Association^13^ (2009), Growing Food & Justice For All Initiative,^14^ and the National Black Food and Justice Alliance.^15^ These groups are working to halt and to reverse what a journalist has called “the great land robbery” of the past century (Newkirk, 2019), in which 98% of African American farmers lost land via a second round of take-over by Whites.

The movement to reclaim our foodways includes many cookbooks, including award-winning ones (Lewis, 1976; Sherman & Dooley, 2017; Tipton-Martin, 2019). Because preventing literacy was among the strategies for oppressing enslaved people, Black chefs largely relied on oral history and experience for cooking (Harris, 2011). What is probably the first African American-authored cookbook appeared in 1881, *What Mrs. Fisher Knows About Old Southern Cooking* (Fisher, 1881). Some White Southerners who learned these foodways from Black people who served them took credit for and published cookbooks with their recipes (Harris, 2011; Wallach, 2019). For Native Americans, recipes were always oral, shared by demonstration and practice. What may have been among the first written collections by a Native person was published in the early 1990s (Hunt, 1992).

The movement includes a growing body of restories like the present paper, including writings about African American (Garth & Reese, 2020; Miller, 2013; Opie, 2008; Penniman, 2018; Reese, 2019; Twitty, 2017, Wallach, 2015, 2019; White, 2018; Williams-Forson, 2006; Zafar, 2019) and Native American foodways (Berzok, 2005; LaDuke, 1999; Mihesuah & Hoover, 2019; Nelson, 2008; Settee & Shukla, 2020). This journal has published a special issue, *Indigenous Food Sovereignty in North America* (Hilchey, 2019). These writings are in addition to a growing number of peer-reviewed, grey, and historical fiction literatures, including books for children (Erdich, 1999–2016; Rhodes, 2013).

#### Example: Facing Down Food Marketing

**Figure 2.**
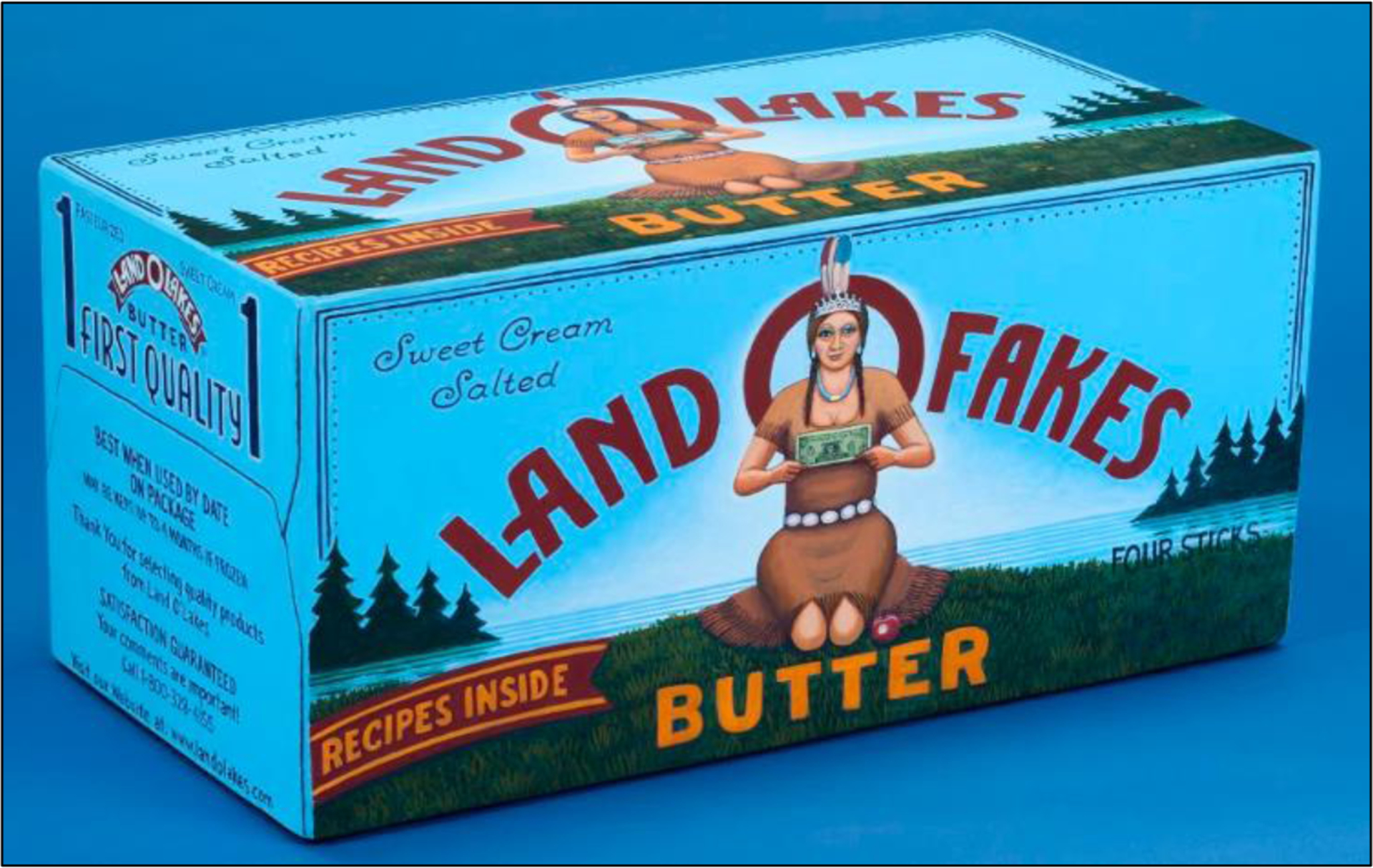
David Bradley, “Land O Bucks, Land O Fakes, Land O Lakes,” 2006. Source: Denver Art Museum.

**Figure 3.**
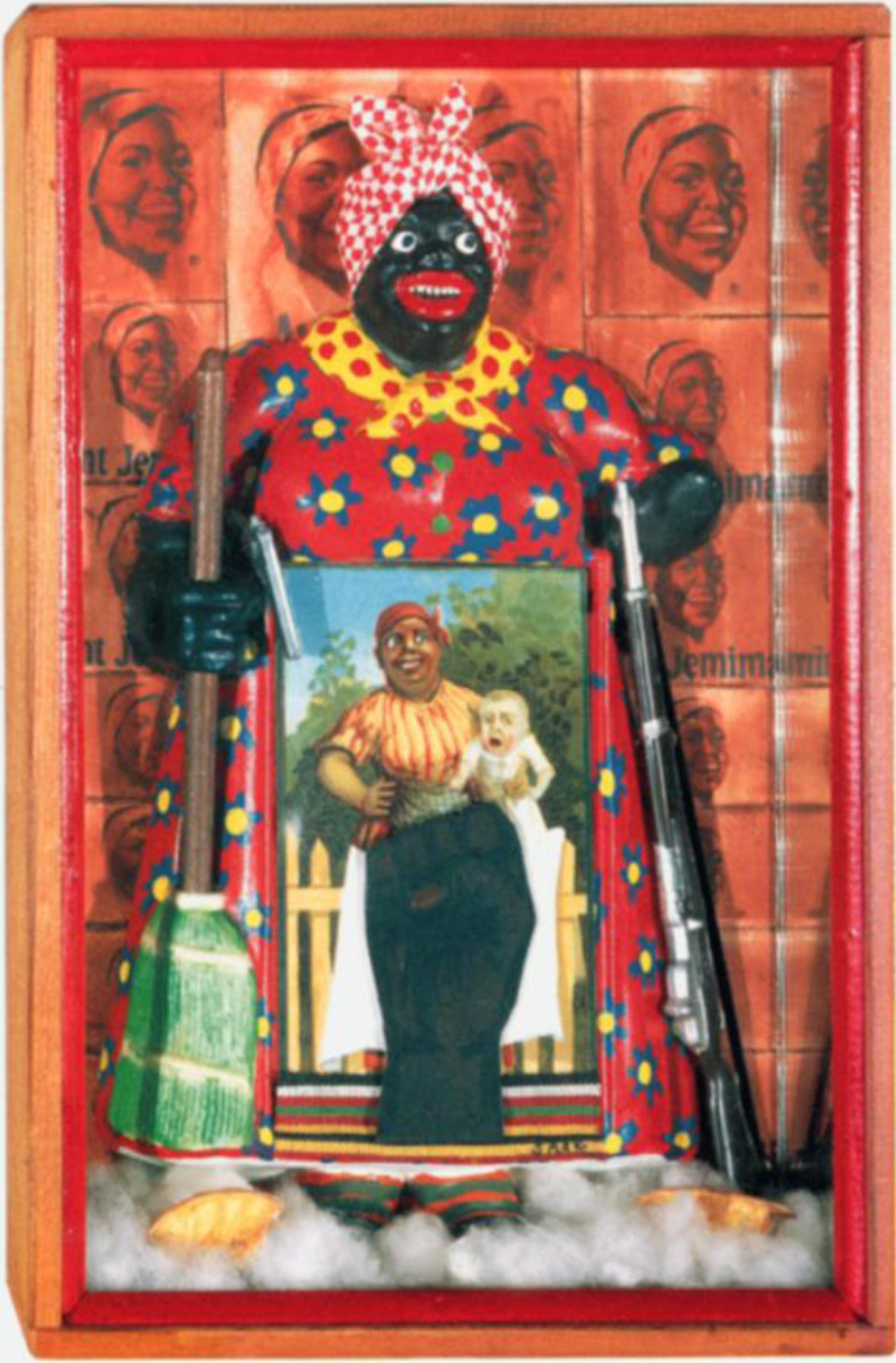
Betye Saar, “The Liberation of Aunt Jemima,” 1972. Source: Berkeley Art Museum.

Anti-racist organizing has included fighting against racist imagery used in marketing and branding, such as by sports teams and food corporations. For example, artists David Bradley and Betya Saar indict the Land O’Lakes and Aunt Jemima marketing imagery, respectively, in the artworks shown in Figures 2 and 3.

Saar wrote about this piece, saying,

I found a little Aunt Jemima mammy figure, a caricature of a black slave, like those later used to advertise pancakes. She had a broom in one hand and, on the other side, I gave her a rifle. In front of her, I placed a little postcard, of a mammy with a mulatto child, which is another way black women were exploited during slavery. I used the derogatory image to empower the black woman by making her a revolutionary, like she was rebelling against her past enslavement.

(Saar, 2016, para. 14)

### Suffocating in a Pandemic (2020)

The oppression and trauma inflicted on our communities for over 400 years has produced enormous health inequities between African and Native Americans and Whites. Whether it is police knees on our necks, wildfire smoke in our lungs, or suffocation by COVID-19, we are fighting for breath.

Native women are more than twice as likely, and Black women more than three times as likely, to die in childbirth as White women (Petersen et al., 2019). Our communities also suffer much higher rates of diabetes, obesity, high blood pressure, child asthma, and other chronic health conditions than Whites (Akinbami et al., 2014; Centers for Disease Control and Prevention, 2005; Porter et al., 2019). This is in part because of the devastation to our food systems and associated historical traumas previously described (Belanger et al., 2020; Gray et al., 2020). We are exposed to more air pollution than White communities. For example, in Minnesota, 91% of communities of color breathe air above risk guidelines, compared with 32% for the state overall (Minnesota Pollution Control Agency, n.d.). We also disproportionately live and/or work in crowded conditions—including in the food industry—that make us more vulnerable to the COVID-19 virus. As a result, our death rates from the pandemic, so far, are about double that of Whites (Laster Pirtle, 2020; Webb Hooper et al., 2020).

The recession caused by COVID-19 is the most unequal in U.S. history (Long et al., 2020). COVID-19 exposed core weaknesses in the dominant industrialized, centralized, globalized and justin-time food system (Hamilton et al., 2020). These weaknesses are in addition to widely known problems that compromise the nutritional capability of future generations by using up resources such as soil, oil, and water. And still there are ongoing threats of the further erasure of cultural food traditions.

However, Native and African American communities also lead the way in building solutions to these problems, finding ways not only to cope, but even to thrive, amid these systemic catastrophes. For example, two women in Wind River Indian Reservation launched a project that provides garden boxes, supplies, and growing lessons via online conference to help people grow their own food (Grow Our Own 307, 2021). The Quapaw Nation’s beef processing facility kept meat in stores while still protecting their workers during the pandemic by prioritizing community safety over speed and profit (Native Business staff, 2020). A community garden in an African American, food-insecure community in Indianapolis quickly pivoted from volunteer growing operations to a youth farm with paid senior workers, to continue providing fresh food to the community while keeping workers safe (Lawrence Community Gardens, 2021). These are merely three of thousands of community-led projects that demonstrate solutions that African and Native American foodways offer for health, equity, and sustainability.

### Knowing and Showing How to Thrive (Our Ancestores to Our Grandchildren)

Our communities grow gardens and farms, preserve food and save seeds, form cooperatives and found food hubs, host and sell at farmers markets, and start soul food and indigenous cafes. We know how to use every part of an animal (Hoover, 2020; Opie, 2008). Some African Americans lead vegan responses to the ethical and environmental travesties of concentrated animal feeding operations (Harper, 2013; Terry, 2009).

Home gardens especially have anchored our family strategies to nurture ourselves. For example, Eastern Shoshone and Northern Arapaho people in Wind River Reservation report (Budowle et al., 2019) that:

A long time ago, if you didn’t have a garden, you didn’t eat.

(p. 153)

When I was growing up my folks had a big old huge garden, and we never went to town, bought candy or anything. When we got hungry, we’d just run out to the garden and get us a turnip or carrots.

(p. 154)

I never knew how to go to the grocery store growing up. We ate everything canned. And now, I’m trying to learn how to do all that stuff after all these years. It is a lot healthier. People were healthier back then.

(p. 155)

LL heard similar stories from African American elders. One recalled, “Besides my mom, other people had their own garden, everybody had a little space…. Back then you didn’t go to the store ‘cause you had your own garden.”

We will close this circle with two examples of historical foodway strategies that could help save our food systems: using good fire and revisioning land access. Our final words point to paradigms and policies to help make these kinds of changes possible. In the face of COVID-19, we need them, for all people, even more than ever (Worstell, 2020).

#### Example: Using Good Fire to Nurture Foodways

For millennia, Indigenous people in North America have intentionally and strategically used controlled burning in forests and prairies to renew foodways, maintaining habitats and life cycles of food and medicine sources. These practices were especially important in the West (Anderson, 2013). They reduced the risk of catastrophic western wildfires, like we are seeing today, that spread uncontrollably and burn everything to the ground.

“Prescribed fire is medicine,” says a research ecologist with Karuk heritage and Yurok family (Cagle, 2019, “Fire is in our DNA,” para. 5). For over a century Whites suppressed these traditional land care practices. Forest and land management policy has been to extinguish fires, any fires, immediately. But recently, as one headline puts it, “To Manage Wildfire, California Looks to What Tribes Have Known All Along” (Sommer, 2020). Ron Goode, tribal chairman of the North Fork Mono, recalls his mother getting in trouble with the fire department for using good fire. He explains, “We don’t put fire on the ground and not know how it’s going to turn out. That’s what makes it cultural burning, because we cultivate … What we’re doing out here is restoring life” (Sommer, 2020, para. 6). As the director of natural resources of the Karuk tribe states, “The solution to the devastating west coast wildfires is to burn like our Indigenous ancestors have for millennia” (Tripp, 2020, para. 14).

#### Example: Proposing 40 Acres

As the Civil War raged, the U.S. developed plans to confiscate land and other property of those who rebelled against the country. In 1861, editors of a Black-run paper noted that when the war ended “there will be four million free men and women and children, accustomed to toil.” They argued that they should be given the confiscated land^16^ with which to rebuild their new lives.

As the war was coming to a close, 20 Black ministers and other lay leaders met with General Sherman and Secretary of War Stanton in Savannah, Georgia, in January 1865. Their spokesman, Reverend Garrison Frazier, who had been enslaved until purchasing his freedom in 1857, said, “The way we can best take care of ourselves is to have land, and turn it and till it by our own labor … and we can soon maintain ourselves and have something to spare. … We want to be placed on land until we are able to buy it and make it our own” (Gates, 2013, para. 12). He suggested that this land be separate from Whites, “for there is a prejudice against us in the South that will take years to get over” (Gates, 2013, para. 12).

Four days later, Sherman issued Special Field Order No. 15, specifying that about 400,000 acres on the coasts of South Carolina, Georgia, and northern Florida were to be confiscated from Confederate traitors to the U.S. and allocated to newly free Blacks to settle and farm, on affordable rent-to-own terms. The area was quickly dubbed the “Sherman Reservation.” The order stated that in the area “the sole and exclusive management of affairs will be left to the freed people themselves” (Sherman, 1865, para. 2).

Soon, 40,000 freed Blacks had settled the land. However, President Johnson rescinded the order in late 1865 and returned the land to White Confederates. Motivated in part by growing claims that the substance of the order had been—to use today’s terms—fake news, a contemporary scholar castigated the aftermath of the order: “The expectations of the blacks were justified by the policies of the Government…rascals took advantage of the expectations to swindle the ignorant freedmen” (Fleming, 2020/1906, p. 1).

Scholars have calculated what wealth these lands would have generated for African Americans had they remained in their hands. Adjusting for inflation and interest, this would be about US$1.6 trillion today, or about US$36,000 for every African American person in the U.S. More importantly, “had the promise of 40 acres been fulfilled, one can readily imagine a completely different U.S. history unfolding over the course of the subsequent century, a history in which race did not intertwine with dense inequalities” (Darity, 2008, p. 661).

#### Providing Paradigms and Policies for Change

Water is life. It takes a village to raise a child. We are all related. Plants and animals are also our relatives. These are paradigms that would lead us to care for the water, soil, air, and all living things that give us life.

We do not mean to romanticize. With racist legacies of poverty, violence, and stress undermining our own communities, such traditional ways are only aspirational for many.

To help reach such aspirations, food policy recommendations come from the Native Farm Bill Coalition and the Movement for Black Lives (M4BL). Building from a report of Indigenous issues and opportunities in the farm bill (Hipp & Duran, 2017), proposals include allowing self-governance of USDA programs by sovereign nations, supporting Tribal departments of food and agriculture, providing relief on farm loans due during the pandemic, recognizing traditional ecological knowledge conservation practices, and including more variety and quantity of traditional foods in assistance programs.

Food policy planks of the M4BL include:

- A right to restored land, clean air, clean water, housing, and an end to the exploitative privatization of natural resources— including land and water. We seek democratic control over how resources are preserved, used and distributed, and do so while honoring and respecting the rights of our Indigenous family.
- Low-interest, interest-free, or federally guaranteed low-interest loans to promote the development of cooperatives (food, residential, etc.), land trusts, and culturally responsive health infrastructures that serve the collective needs of our communities.
- Protections for workers in industries that are not appropriately regulated, including domestic workers, farmworkers, and tipped workers, and for workers—many of whom are Black women and incarcerated people—who have been exploited and remain unprotected (M4BL, n.d.)

#### Now What?

Everything we discussed in the *Reclaiming and Restoring* section provides some direction and reasons for hope for our communities and our foodways. For example, in *McGirt v. Oklahoma*, the Supreme Court finally has directed the government to enforce at least one of the over 500 treaties it has broken. In the face of police brutality, the Black Lives Matter movement has mobilized people of all racial groups for justice for all people of color. Even Wyoming, by many measures the most politically conservative state in the U.S., had marches in every town we can name, some continuing into fall 2020. Under pressure from this work, the corporations that contrived Aunt Jemima, Uncle Ben, and the Land O’Lakes Indian maiden are retiring such co-optations, as are some major sports teams. Reparations for enslavement are on the table in serious policy discussions for the first time (Ho, 2020).

Nevertheless, the scale and scope of the brutality and theft in our story dwarf the steps taken and proposed for repairing damage and redressing injustices. For example, as ML reports about the Land O’Lakes victory, people are saying they took away the Indian and kept the land. Native and African American communities fight despair with nourishment and attend to the work to restore, reclaim, and renew our traditional foodways. A Rarámuri scholar of Indigenous foodways says that “eating is not only a political act but also a cultural act that reaffirms one’s identity and worldview” (Salmón, 2012, p. 8) each time one sits down to eat a culturally rich food.

We close with the thoughts of two elders we learned from during our research:

Being able to control what we eat is also like saying we have control over our lives, ourselves.

—African American food expert and elder

I hope this is a revolution.

—Northern Arapaho food expert and elder

## Figures and Tables

**Table 1.** Summary of Key Eras in African American and Native American Foodways, 1500s–2020

Era	African American Foodways	Native American Foodways
**Living** most of our story (origins to 1500s and 1619) West African foodways Native American foodways	Tropical coastal climate. Rice, sorghum, yam, black-eyed pea agriculture. Add corn, peanuts, and cassava from North American imports in 1500s. Gather, fish, small-game hunt, animal husbandry. Vegetable-centered stews, flavored with meat or fish and often peppers, served with starch staples.	6 foodways adapted to climates. Gather, nurture wild foods, fish, hunt. Maize, bean, squash agriculture in East and Southwest. Bison in Great Plains, wild rice in Great Lakes, maple syrup cultivated in New England, fish on coasts (especially salmon in Northwest), pine nuts in Great Basin.
**Enduring** enslavement, epidemics, encroachment, and invasion (1500s/1619 to 1700s) Dying Suffering enslavement Fighting to retain traditional land and foodways Allying and being divided	Kidnapping and enslavement devastated West African populations by about 20%. Many died on Middle Passage. Instant severance from food and lifeways. Employ skills and seeds to grow West African and local options. Survive on corn and pork rations; gardens, foraging and small-game hunting as “owners” allowed. Endure brutality, including rape and whippings.	Smallpox epidemics killed more than any other disease or war with invaders, sometimes entire tribes. Gradual shifts in food and lifeways. Teach English invaders foodways to help them survive, who encroach with colonizers and cattle. Enslaved by Spanish invaders in Florida and Southwest; Pueblo Revolt in 1680. Nations in East negotiate and fight for land. Horses + guns aid and expand bison-based foodways.
**Building** the U.S. (to 1865 and turn of the century) Putting soul into food Negotiating for survival Transitioning to next phases of oppression	U.S. economy builds on enslaved food and fiber labor. Create survival foods such as chitlins. Adapt West African stew traditions and to “owner” preferences to invent soul foods such as peach cobbler, sweet potato pie, and collards with ham hocks. Establish Underground Railroad to facilitate escape from slavery.	U.S. builds on stolen land. Forced onto reservations by starvation (including U.S. bison massacre) and massacres. Endure cultural genocide tactics, e.g., Dawes Act to reduce reservations, boarding schools, ration foods (invent frybread out of them), and imposition of European agriculture. Negotiate over 500 treaties with U.S., which breaks all of them.
**Surviving** White American supremacy (to 1950s) Reestablishing dislocated lives E.g.: Facing food marketing Being fed rations and shame	Enslavement ends for 4 million of us. Supremacy does not. Establish flourishing towns, governments and neighborhoods, then crushed by White violence. Become sharecroppers and servants as only options in a Jim Crow–ruled Southeast. Great migration out of the South, though racism still rules the nation. Foods co-opted as White southern food and used for corporate marketing, and yet soul food demonized.	Rebuild lives with fewer than 250,000 of us left, mainly on reservation; nowhere near homelands and associated foodways for most. Lose a total of 2/3 of allotted land by 1935 due to Dawes Act. Suffer destitution, poverty and misery; scratch out foodways with gardening, farming, some hunting, and food rations. Face additional assimilation strategies, including continued boarding schools and Indian Relocation Act of 1956.
**Reclaiming and restoring** (1960s–2000s) Organizing with food and foodways E.g.: Facing down food marketing	Found the civil rights movement, from Martin Luther King Jr. (MLK) to Black Lives Matter (BLM). Fight and win reparations case against USDA. Establish land tenure and food justice organizations and initiatives. E.g., fight and win against racist appropriations in industrial food marketing.	Build a rights movement, starting in urban areas. Fight and win reparations case against USDA and honoring of an 1863 treaty establishing half of Oklahoma as Creek land after removal from Southeast. Establish food sovereignty and land tenure organizations and initiatives. E.g., fight and win against some racist appropriations in industrial food marketing and sports.
**Suffocating** in a pandemic (2020)	We can’t breathe. Have knees on our necks; disproportionate air pollution and COVID in our lungs. Have high rates of pre-existing conditions from legacies of supremacy outlined here. Disproportionately do front-line food and medical service jobs, exposing us to the virus. Dying from COVID at 2 to 3 times the rate of Whites.	We also can’t breathe. Suffer policy brutality; disproportionate air pollution and COVID in our lungs. Have high rates of pre-existing conditions from legacies of supremacy outlined here. Dying from COVID at 2 to 3 times the rate of Whites. Reservation-based Nations provide leading public health responses in testing, isolating, and tracing.
**Knowing and showing** how to thrive (our ancestors to our grandchildren) E.g.: Using good fire to nurture foodways E.g.: Proposing 40 acres Providing paradigms and policies for change Now what?	Retain and restore local foodways including gardens, markets, cooperatives, seed saving, soul food cooking. Propose Movement 4 Black Lives policy platform. Offer human relationship–centered and collective paradigms for foodways that nourish.	Retain and restore traditional foodways including three sisters gardening, cultural burning, gathering, establishing bison herds, preparing traditional foods and medicines. Propose Native Farm Bill Coalition policies. Offer relational paradigms that center connections between humans, other living beings, earth, our ancestors, and our descendants.

## Footnotes

1
https://nativeamericanagriculturefund.org/

2
https://nativeamericanagriculturefund.org/2020-grantees/

3
https://www.welrp.org

4
https://www.dbcfsn.org

5
https://www.firstnations.org

6
https://www.indianag.org/

7
https://iltf.org/

8
https://indigenousfoodandag.com/

9
https://nativefoodalliance.org/

10
http://www.ncai.org/initiatives/partnerships-initiatives/food-sovereignty

11
https://www.nationalblackfarmersassociation.org/

12
http://saafon.org/

13
https://www.blackurbangrowers.org/

14
https://www.facebook.com/growingfoodandjustice/

15
http://www.blackfoodjustice.org/

16 This land had been taken in the previous centuries from the Creeks and Seminoles, who were killed, forced to move to reservations in what is now Oklahoma, or died from privations on the journey.
